# Phylogenetic diversity and genotypic complexity of H1N1 subtype swine influenza viruses isolated in Mainland China

**DOI:** 10.1186/1743-422X-9-289

**Published:** 2012-11-26

**Authors:** Yizhi Liu, Jing Wang, Jun Ji, Shuang Chang, Chunyi Xue, Jingyun Ma, Yingzuo Bi, Qingmei Xie

**Affiliations:** 1 College of Animal Science, South China Agricultural University, Guangzhou, 510642, China; 2 Guangdong Wen’s Foodstuffs Group Co. Ltd, Yunfu, 527439, China; 3 Department of Animal Science, Michigan State University, East Lansing, MI, 48824, USA; 4 State Key Laboratory of Biocontrol, College of Life Sciences, Sun Yat-Sen University, Guangzhou, 510006, China

## Abstract

**Background:**

After the occurrence of 2009 pandemic H1N1, close attention has been paid to the H1N1 subtype swine influenza viruses (H1N1 SIV) by scientific communities in many countries*.* A large-scale sequence analysis of the NCBI Influenza Virus Resource Database on H1N1 SIVs submitted primarily by scientists in China during 1992 to 2011 was performed. The aims of this study were to elucidate the genetic and evolutionary characteristics of H1N1 SIVs, to identify and unify the lineages and genetic characteristics of the H1N1 SIVs isolated in mainland China.

**Results:**

Most of the strains were isolated during the period of 2008 to 2010 from Guangdong and Shandong provinces, China. Based on the phylogenetic and genotypic analyses, all of the H1N1 SIV strains can be classified into 8 lineages and 10 genotypes. All strains were of the characteristics of low pathogenic influenza viruses. The viruses of different lineage are characterized with different amino acid residues at the receptor-binding sites. Viruses containing PB2 genes of the classical swine, early seasonal human and recent seasonal human lineage might be more infectious to human. Some genotypes were directly related with human influenza viruses, which include strains that harbored genes derived from human influenza viruses.

**Conclusions:**

Phylogenetic diversity and complexity existed in H1N1 SIVs isolated in mainland China. These H1N1 SIV strains were closely related to other subtype influenza viruses, especially to human influenza viruses. Moreover, it was shown that, novel lineages and genotypes of H1N1 SIVs emerged recently in mainland China. These findings provided new and essential information for further understanding of the genetic and evolutionary characteristics and monitoring the H1N1 SIVs in mainland China.

## Background

Influenza is a very important viral respiratory disease causing outbreaks yearly in tropical and subtropical countries with low morbidity but high mortality and has been posing a threat to economy and public health [1]. Influenza A viruses have been isolated from humans and a number of animals including birds, dogs, seals, horses, and swine [2].

To date, based on hemagglutinin (HA) and neuraminidase (NA) of the two surface glycoproteins [2,3], 16 subtypes of HA and 9 subtypes of NA have been found in influenza A viruses [4-6]. Swine influenza was caused by influenza A virus firstly isolated in 1930 in North American by Shope [7]. H1N1, H3N2 and H1N2 that include classical swine H1N1, European avian-like H1N1, human-like H3N2, reassortant H3N2, and various genotype H1N2 viruses are the three main subtypes of influenza viruses are circulating in the swine population worldwide [8-11].

Swine influenza viruses cause significant economic losses in animal husbandry, human disease [12], and occasionally give rise to human pandemics [13]. The 2009 pandemic H1N1, a new swine-origin influenza A (H1N1) virus, caused the latest human pandemic [14,15]. After the occurrence of the 2009 pandemic H1N1, the virus was repeatedly introduced back into pigs in many countries [16-18].

With the swine influenza virus movement increasing, it raised hardness to predict the evolutionary consequences, and demand consideration given an increasingly globalized future [19]. It was reported that reconstructing the origins of pandemic have been hampered as a result of lacking systematic and longitudinal influenza surveillance in pigs [13]. Most existing swine data were derived from opportunistic samples collected from diseased pigs in disparate geographical regions, not from prospective studies in defined locations. Hence the evolutionary and transmission dynamics of swine influenza viruses are poorly understood [19].

Since the first isolation of a human influenza virus in 1933, 1957 and 1968 pandemics originated in China [20]. Findings have contributed to the hypothesis that China serves as an epicenter of pandemic influenza viruses throughout history [21]. Since the first report of H1N1 SIVs in China in 1992 [22], several studies of H1N1 SIVs prevalence and genetic have been carried out. China was the biggest pork producer in the world, as the pork industry continued to develop, swine influenza virus became prevalent in mainland China especially in recent years. We performed a large-scale sequence analysis of the NCBI Influenza Virus Resource Database on H1N1 SIVs isolated in mainland China from 1992 to 2011, to comprehensively elucidate the genetic and evolutionary characteristics of H1N1 SIVs in mainland China, and attempt to identify and unify all lineages and genotypes of H1N1 SIVs in mainland China.

## Results

### Strains differences based on separation time point and region

In this study, 94 strains and 712 segments of H1N1 SIVs in mainland China (Additional file 1: Table S1) were obtained from the NCBI Influenza Virus Resource Database (http://ncbi.nlm.nih.gov/genomes/FLU/FLU.html) till December 27, 2011.

Reported strains in GenBank were mostly isolated in recent years especially in 2008, 2009, and 2010, in Guangdong and Shandong province (Table 1). As pigs raised in increasing numbers continually in many provinces of mainland China, H1N1 SIVs might become more prevalent in the nation.

**Table 1:** Regularity of the H1N1 subtype swine influenza viruses in China mainland from 1992 to 2011 in the separation time and region

	**Total number**	**Year**										
		**1991**	**2001**	**2002**	**2004**	**2005**	**2006**	**2007**	**2008**	**2009**	**2010**	**2011**
Beijing	4	2							2			
Fujian	3							1	2			
Guangdong	56		2	1		2	7		4	14	26	
Guangxi	1											1
Henan	1						1					
Hubei	5									5		
Jiangsu	2											2
Liaoning	1						1					
Nanchang	4										4	
Shandong	12								9	3		
Shanghai	3					3						
Tianjin	1				1							
Zhejiang	1							1				

### Phylogenetic analysis of H1N1 subtype swine influenza viruses in mainland China from 1992 to 2011

To elucidate the genetic and evolutionary characteristics of H1N1 SIVs in mainland China from 1992 to 2011, the genomes of the viruses were analyzed along with the reference sequences available in GenBank, consisting of viruses isolated from swine, avian, and human. Eight viral gene segments of the viruses were aligned and analyzed phylogenetically.

**Figure 1:**
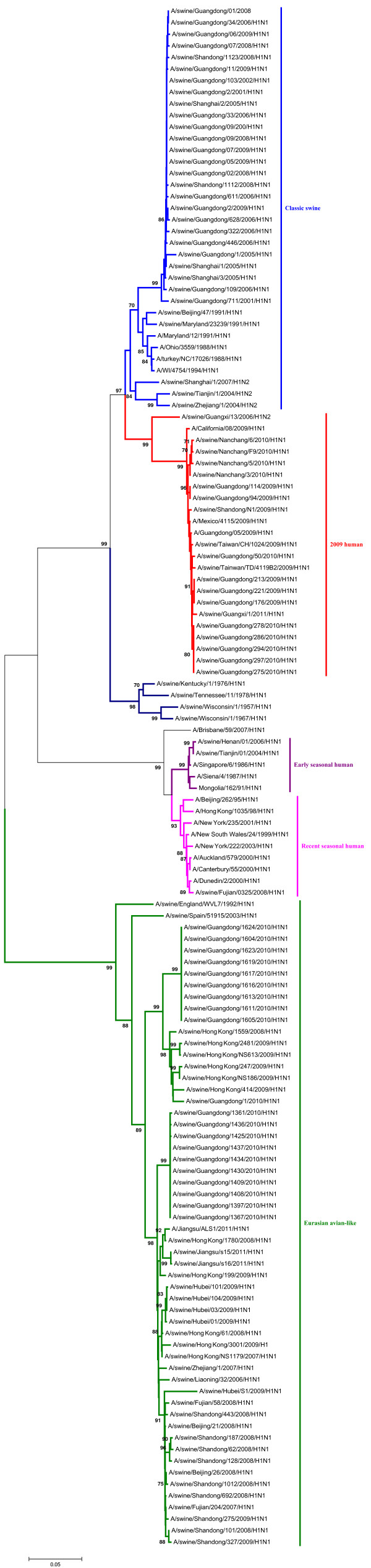
**Phylogenetic tree of the HA gene of the H1N1 subtype swine influenza viruses. **The unrooted phylogenetic trees were generated by the neighbor-joining method using MEGA 5.0 software. Bootstrap values were calculated out of 1000 replicates and only bootstrap values of ≥70% were shown. Different lineages were indicated by different colors.

**Figure 2:**
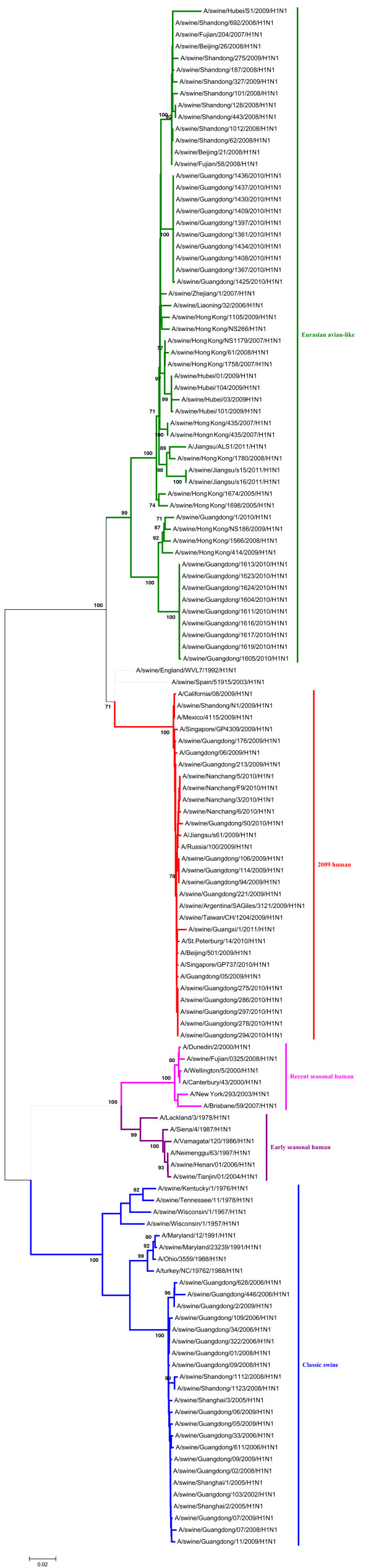
**Phylogenetic tree of the NA genes of the H1N1 subtype swine influenza viruses. **The unrooted phylogenetic trees were generated by the neighbor-joining method using MEGA 5.0 software. Bootstrap values were calculated out of 1000 replicates and only bootstrap values of ≥70% were shown. Different lineages were indicated by different colors.

In the phylogenetic trees of the HA and NA gene of the H1N1 SIVs (Figures 1, 2), it indicated that the HA and NA gene of H1N1 SIVs in mainland China mainly fall into five lineages, the classical swine (CS) lineage, the early seasonal human (ESH) lineage, the recent seasonal human (RSH) lineage, the Eurasian avian-like (EA) lineage, and the 2009 human (2009H) lineage.

**Figure 3:**
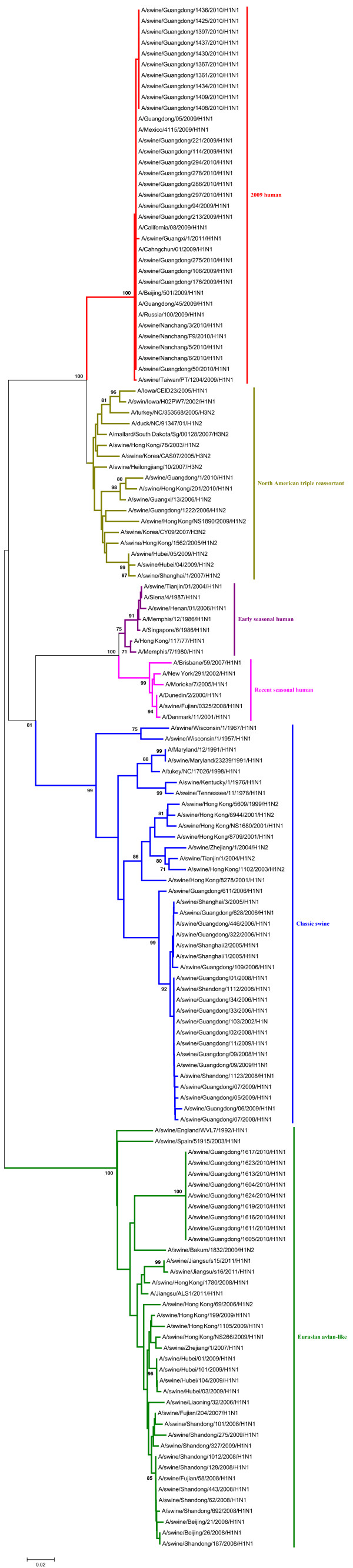
**Phylogenetic tree of the PB2 genes of the H1N1 subtype swine influenza viruses. **The unrooted phylogenetic trees were generated by the neighbor-joining method using MEGA 5.0 software. Bootstrap values were calculated out of 1000 replicates and only bootstrap values of ≥70% were shown. Different lineages were indicated by different colors.

**Figure 4:**
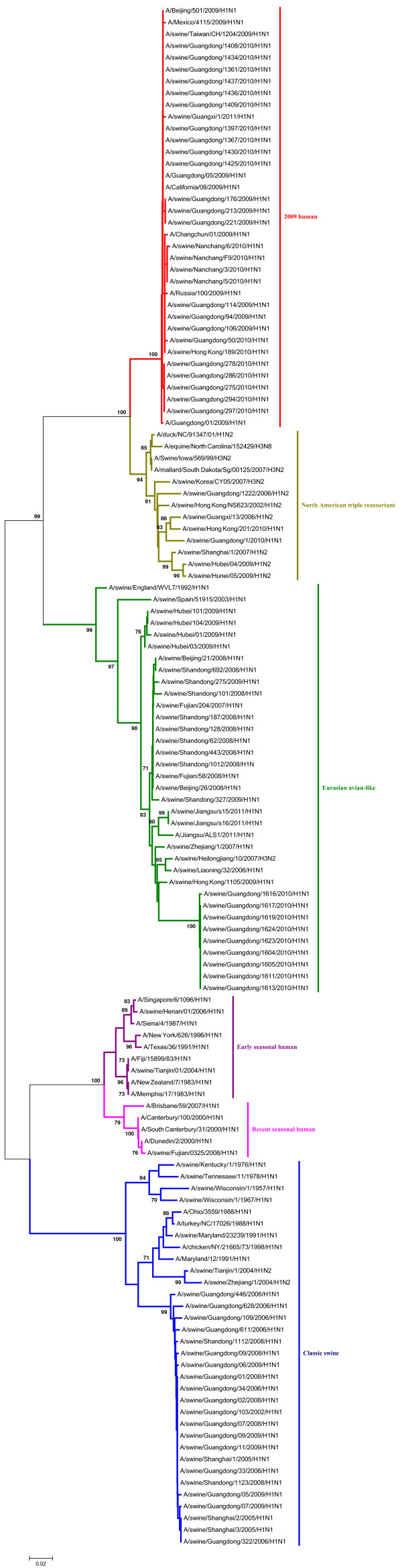
**Phylogenetic tree of the PA genes of the H1N1 subtype swine influenza viruses. **The unrooted phylogenetic trees were generated by the neighbor-joining method using MEGA 5.0 software. Bootstrap values were calculated out of 1000 replicates and only bootstrap values of ≥70% were shown. Different lineages were indicated by different colors.

**Figure 5:**
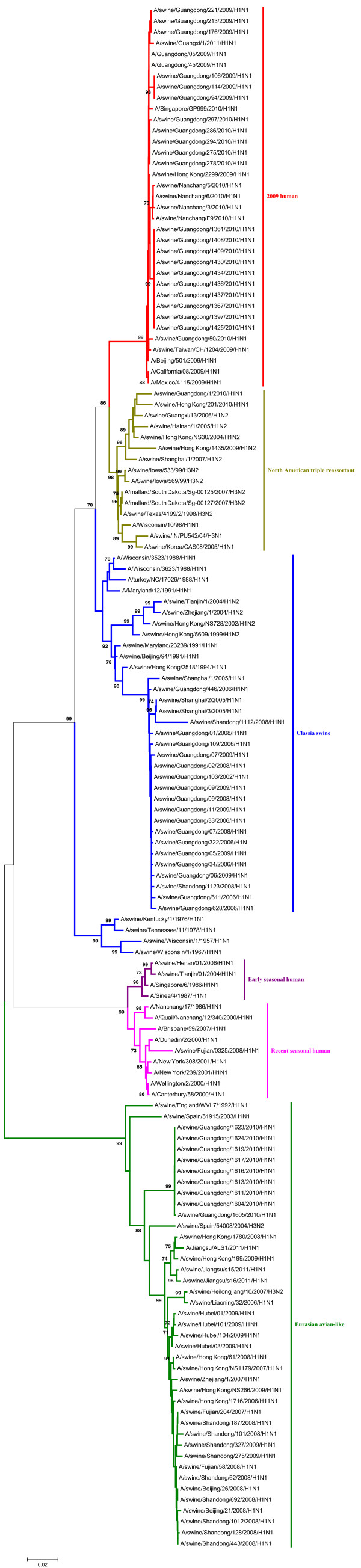
**Phylogenetic tree of the NP genes of the H1N1 subtype swine influenza viruses. **The unrooted phylogenetic trees were generated by the neighbor-joining method using MEGA 5.0 software. Bootstrap values were calculated out of 1000 replicates and only bootstrap values of ≥70% were shown. Different lineages were indicated by different colors.

**Figure 6:**
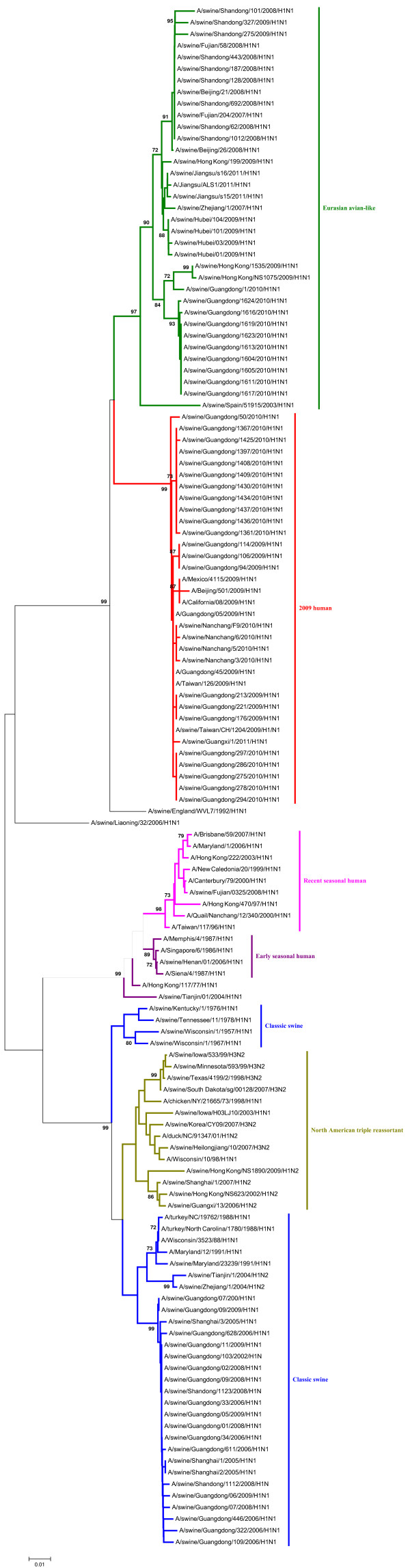
**Phylogenetic tree of the M genes of the H1N1 subtype swine influenza viruses. **The unrooted phylogenetic trees were generated by the neighbor-joining method using MEGA 5.0 software. Bootstrap values were calculated out of 1000 replicates and only bootstrap values of ≥70% were shown. Different lineages were indicated by different colors.

Phylogenetic analyses of the PB2, PA, NP and M gene showed that those could be separated into six major subgroups (Figures 3, 4, 5, 6), including the CS lineage, the North American triple reassortant (TR) lineage, the EA lineage, the 2009H lineage, the ESH lineage, and the RSH lineage.

**Figure 7:**
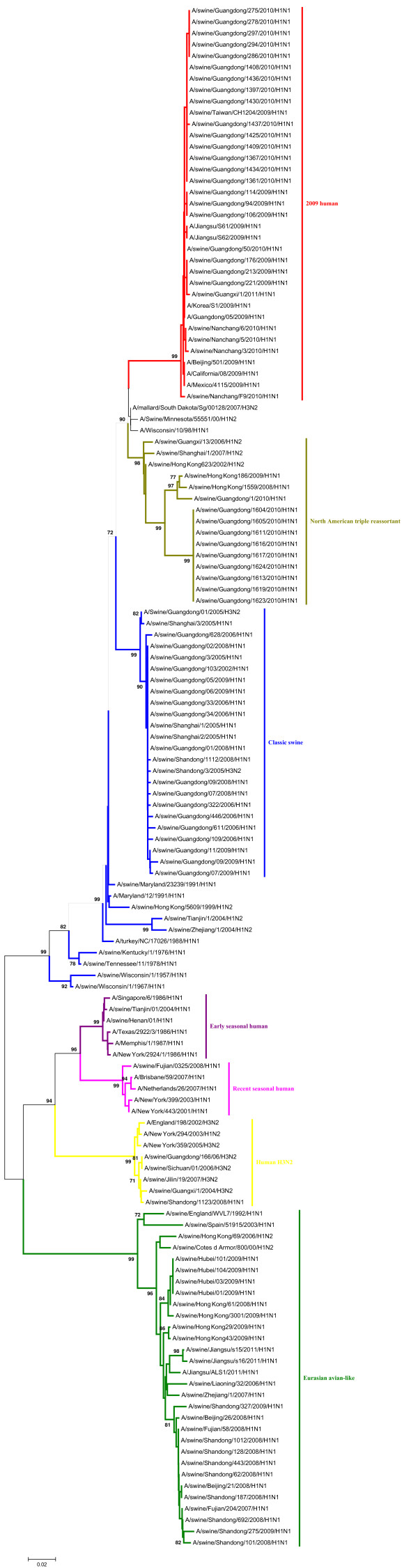
**Phylogenetic tree of the NS genes of the H1N1 subtype swine influenza viruses. **The unrooted phylogenetic trees were generated by the neighbor-joining method using MEGA 5.0 software. Bootstrap values were calculated out of 1000 replicates and only bootstrap values of ≥70% were shown. Different lineages were indicated by different colors.

A phylogenetic analysis of the NS gene showed that all of the NS genes from these H1N1 SIVs could be divided into seven lineages (Figure 7), respectively, which were the CS lineage, the TR lineage, the EA lineage, the 2009H lineage, the ESH lineage, the RSH lineage, and the human H3N2 (H3N2H) lineage.

**Figure 8:**
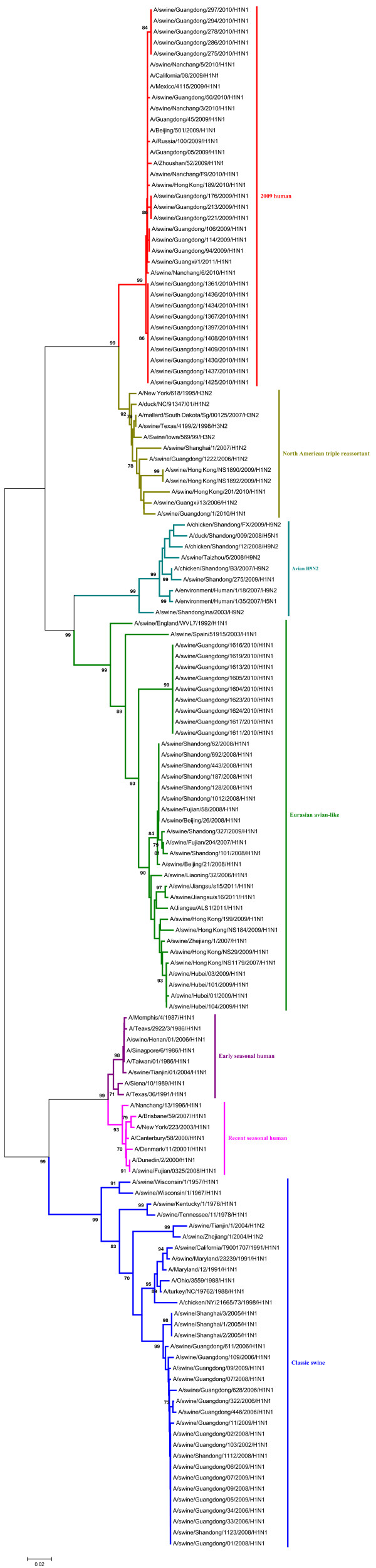
**Phylogenetic tree of the PB1 genes of the H1N1 subtype swine influenza viruses. **The unrooted phylogenetic trees were generated by the neighbor-joining method using MEGA 5.0 software. Bootstrap values were calculated out of 1000 replicates and only bootstrap values of ≥70% were shown. Different lineages were indicated by different colors.

As shown in the phylogenetic tree of the PB1 genes, clear divisions of each of these genes into different lineages exited, including the CS lineage, the TR lineage, the EA lineage, the 2009H lineage, and the avian H9N2 (H9N2A) lineage (Figure 8).

The detailed analysis of the genetic relationship between H1N1 SIVs and human influenza viruses displayed that the 2009H lineage and the TR lineage were located in the sister lineage in the phylogenetic trees of the PB2, PB1, PA, NP and NS gene, while the 2009H lineage was in the sister lineage with the EA lineage in the phylogenetic trees of the NA and M genes, similarly with previous research [15]. In the phylogenetic trees of the PB2, PB1, PA, HA, NP and M gene, the CS, ESH and the RSH lineages were in the sister lineage, while the EA lineage was in the sister lineage with the RSH, ESH and H3N2H lineages in the phylogenetic tree of the NS gene. The gene segments of REH, ESH, 2009H, and H3N2H lineage derived from recent human influenza viruses, early human influenza viruses, 2009 human pandemic influenza viruses, and human H3N2 influenza viruses respectively. And the genes of H9N2A lineage derived from avian H9N2 influenza viruses. Those should be obtained special attention.

Interestingly, through the panorama view of the resulting phylogenetic trees, we found that early human influenza virus genome segments appeared in the CS lineage. And a human influenza virus strain (A/Jiangsu/ALS1/2011/H1N1) appeared in the EA phylogenetic tree, indicating that the avian-origin European H1N1 SIVs remain endemic in swine and have retro-infected humans after circulating through swine [23].

These findings further highlighted the phylogenetic diversity and complexity of the H1N1 subtype swine influenza viruses, and showed that H1N1 subtype swine influenza viruses were related to other subtype influenza viruses in mainland China.

### Panorama genotypic diversity of H1N1 subtype swine influenza viruses in mainland China

**Figure 9:**
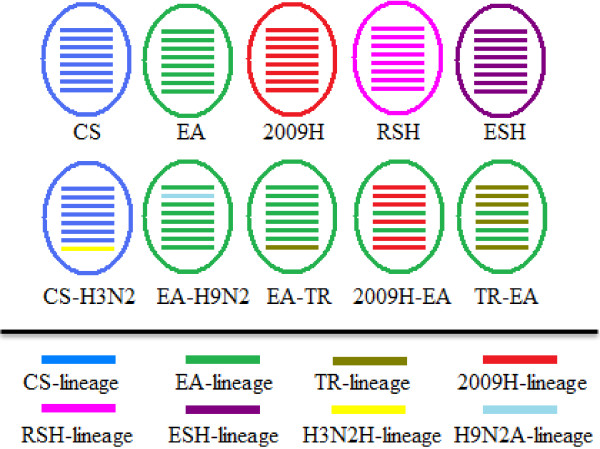
**The genotypes of H1N1 subtype swine influenza viruses in China mainland from 1992 to 2011. **The eight gene segments (horizontal bars staring from the top) were PB2, PB1, PA, HA, NP, NA, M and NS genes, and were indicated in same color with representative viruses for each lineage.

Taken together, on the basis of the phylogenetic analyses of all eight gene segments of H1N1 SIVs isolated from pigs in mainland China from 1992 to 2011, the viruses in the present study could be divided into 10 genotypes, which were shown in Figure 9. The genetic composition of the genotypes of CS, EA, 2009H, RSH, and ESH series contained the same segments origin respectively. Of these genotypes CS-H3N2H, EA-H9N2A, EA-TR, 2009H-EA, and TR-EA included the distinct gene sources.

The genotypes of 2009H, ESH, RSH, CS-H3N2H and 2009H-EA were directly related to human influenza viruses, including strains harbored genes derived from human influenza viruses. The eight gene segments of genotype RSH, ESH, and 2009H derived from recent human influenza viruses, early human influenza viruses, and 2009 human pandemic influenza viruses respectively. Genotype CS-H3N2, such as A/swine/Shandong/1123/2008/H1N1, its NS gene originated from human H3N2 influenza viruses, the remaining segments from the classic swine influenza viruses. NS gene of EA-TR genotype viruses belonged to the North American triple reassortant influenza viruses, with other genes derived from the Eurasian avian-like influenza viruses. Genotype EA-H9N2A represented that virus recombinant PB1 gene from avian influenza viruses, remaining genes originated from the Eurasian avian-like influenza viruses. The 2009H-EA genotype strains appeared to evolve from the Eurasian avian-like influenza viruses by recombinant HA, NA gene, and the internal genes from the 2009 human influenza viruses. HA, M, and NA gene of TR-EA genotype strains belonged to the Eurasian avian-like influenza viruses, the other genes derived from the North American triple reassortant viruses.

These findings showed that novel genotypes of H1N1 SIVs were existed in mainland China, and further revealed the diversity and complexity of H1N1 SIVs in mainland China.

### Key site analyses

To investigate the characteristics of different lineages (CS, TR, EA, 2009H, ESH, RSH, H3N2H, and H9N2A) of the H1N1 SIVs, the amino acid sequences of the HA and PB2 genes were also aligned (Table 2).

**Table 2:** The analysis of amino acid sequences of the HA and PB2 genes

**Lineages**	**Cleavage sites**	** HA**		**PB2**
		**222**	**226**	**627**
CS	PSIQSR↓G	G	Q	K
TR	PSIQSR↓G	/	/	E
EA	PSI/VQSR↓G	E/K	L/Q	E
ESH	PSIQSR↓G	G	Q	K
RSH	PSIQSR↓G	G	Q	K
2009H	PSIQSR↓G	D	Q	E

The HA protein was responsible for binding to receptors on host cells and initiating infection, and it was also the principal target of the host’s immune system [2,24,25]. Molecular analysis of the haemagglutinin gene was essential to monitor the fit to the vaccine strain, as well as variations of the gene that may alter the pathogenicity of circulating virus strains.

Based on the sequence alignment, there were no basic amino acids insertion at the HA cleavage sites. Most of the strains of eight lineages contained an amino acid motif PSIQSR↓G at the HA cleavage sites, which met the characteristic of low pathogenic influenza viruses.

The substitution of aspartic acid (D) to glycine (G) at position 222, D222G (225 in N2 numbering) in HA1 subunit confered viral affinity for α2-3/α2-6-linkage specificity,enabling the variant protein to bind to both receptors [26]. Strains belonged to the 20009H lineage possessed D at the position 222, while CS, ESH, RSH lineage strains possessed G. Interestingly, the EA lineage strains possessed E or K at the position 222.

Viruses which contained L226 had a higher affinity for sialic acid α2,6-galactose (SAα2, 6Gal) and a higher infectivity level for primary swine and human respiratory epithelial cells, whereas viruses contained Q226 had lower SAα2,6Gal affinity and lower infectivity levels for both types of cells [27]. Three strains (AEH42763, AEH42796, ADX96236) of EA lineage strains possessed L at the position 226, while the strains of other lineages possessed Q at the position 226. Different lineage viruses had the different amino acid at the receptor-binding sites, which suggested that they might have different host range.

Polymerase played a critical role in the adaptation of avian influenza viruses to mammalians. Avian influenza viruses have E at 627 position of PB2, whereas all human viruses (H1N1, H2N2, and H3N2) have K [28]. Additionally, the mutation E627K was reported to be responsible for host range, tissue tropism and increased virulence of avian viruses in mammals [29,30]. All of the strains of CS, ESH, and RSH lineage possessed K at the position 627, while the strains of other lineage possessed E at the position 627. It was further revealed that PB2 genes of the CS, ESH, and RSH lineage were closely related to human influenza viruses, PB2 genes of the EA, TR, and 2009H lineage were closely related to avian influenza viruses.

## Discussion

Different host species displayed differing virus-binding receptors with preferences for either Neu5Aca2-3Gal or Neu5Aca2-6Gal epitopes [24,31]. Both Neu5Aca2 -3Gal or Neu5Aca2-6Gal receptors existed in the tracheal epithelium cells in pigs, preferred by avian influenza viruses and human influenza viruses, respectively [32,33] indicated that pigs could infect with swine, human, and avian viruses. Therefore, it suggested pigs could act as intermediate hosts, or mixing vessels, for the generation of genetically reassortant viruses with human pandemic potential.

Viruses sequences download from GenBank were mostly isolated in recent years. The relative strains were mainly isolated and reported in Guangdong and Shandong provinces. Most existing swine data were derived from opportunistic samples collected from diseased pigs in disparate geographical regions, not from prospective studies in defined locations [18]. Although the sampling bias might be another reason, pigs were raised in increasing numbers continually in many provinces in mainland China, especially in Guangdong and Shandong province. Meanwhile, swine influenza virus might become prevalent in the nation. To a certain extent, it showed that swine influenza virus became prevalent in mainland China in recent years.

A first study to characterize the evolution of complete genomes of influenza A H3N2, H1N1 and H1N2 isolates from Europe from 1999 to 2006, indicated that H1N1 SIVs had been evolving and might be likely to be the prevalent strain again. And more precise knowledge about the circulating strains might be help for predicting the following season strains [34]. A historical record of the genetic and antigenic evolution of swine influenza viruses in European, and described the transmission of European swine influenza viruses from pigs to other animal species and to humans, together with the factors that limit inter-species transmission [35].

As the classification of lineages to study the influenza viral ecology, epidemiology, and evolution was of great importance, so in our study, we firstly analyzed the allover H1N1 SIVs sequences in mainland China from the GenBank and integrated the information from the publicly available sequences, identified and unified all the lineages and genotypes of H1N1 SIVs in mainland China. Recently, a study has quantified the epidemiological, genetic and antigenic dynamics of swine influenza A viruses in Hong Kong using a data set of more than 650 swine influenza A viruses isolates and more than 800 swine sera from 12 years of systematic surveillance in this region, supplemented with data stretching back 34 years. And their results showed that one reason for lineage change might be a competitive advantage of EA over CS and TRIG viruses [18].

Classical H1N1 swine influenza viruses, co-circulating with H3N2 swine influenza viruses, were most prevalent for a long time. However, since the Asian avian influenza virus-like H1N1 influenza viruses had been isolated from pigs in 1993 and had circulated with classical H1N1 viruses in previous studies [36-38]. In 2007–2008, European avian-like H1N1 viruses were also detected in pigs in China [39,40]. Similar to the H9N2 influenza viruses [41], the phylogenetic diversity and complexity also existed in H1N1 SIVs in mainland China. The knowledge of its diversity and complexity was still inadequate, and had not attracted much attention. Our study identified and unified all the lineages and genotypes of H1N1 subtype swine virus in mainland China, and provided the essential information supporting for further study of H1N1 subtype swine influenza viruses in Mainland China.

Influenza A viruses genetic reassortments, that might generate pandemic strains of influenza A virus, occur when two or more strains of influenza virus co-infect the same cell, especially from different host as the segmented nature of the RNA genomes [42,43]. Study has shown that reassortments between EA and triple-reassortant swine viruses do occur and establish in swine as stable lineages in swine [18]. Recombinant was a process that was a frequent generation of new reassortant but had only a few survival and persistence [44]. Reassortment and antigenic change were linked [18] which has been described in North America CS viruses after the events that generated the triple-reassortant swine viruses [8]. These similar events have also been reported in human influenza [45]. Novel lineages and genotypes of H1N1 SIVs appeared in mainland China, raising the possibility of generating novel H1N1 SIVs with the potential to infect human.

Influenza virus pandemic was considered inevitable. In the early years, studies had focused on the emergence of the next pandemic in China [46-49]. China was the biggest pork producer in the world, almost all of its 50 million metric tons of production in 2010 (half of all the pork in the world) was consumed domestically. Most of the facilities of the pig farm in mainland China cannot provide sufficient temperature control, resulting in pigs susceptible to respiratory diseases, but it has not been given sufficient attention. Furthermore, increasing pig movements, and human, swine influenza viruses co-existing in swine herds made the swine influenza viruses diversity and offered more opportunities to generate reassortment viruses with the potential to infect humans.

Generally, lowly concerning to low pathogenic influenza viruses raise made these strains might have a greater opportunity to become widespread. As amino acid position 222 and 226 was in the receptor binding cavity, changes can potentially influence receptor binding of the influenza virus and host range. Three strains (AEH42763, AEH42796, ADX96236) of EA lineage possessed L at the position 226, might had a higher affinity for sialic acid α2, 6-galactose (SAα2, 6Gal) and a higher infectivity level for primary swine and human respiratory epithelial cells. The strains of other lineages possessed Q at the position 226 might had lower SAα2, 6Gal affinity and lower infectivity levels for both types of cells. A possible correlation of D222G substitution in HA subunit of 2009H viruses with severe clinical outcome was observed [50]. So strains of 2009H, CS, ESH, and RSH lineage, which possessed D222G, should be received special attention. The clinical significance was still unclear about the other substitutions at the same position, EA strains possessed E222 K. The E627K substitution was observed to enhance virulence and viral replication in mice and other mammals [27,51,52]. Our study suggested that H1N1 SIVs contained PB2 genes of the CS, ESH, and RSH lineage might infect human easily.

Special attention and close supervision should be got raised on the genotypes of 2009H, ESH, RSH, CS-H3N2 and 2009H-EA, which were directly related to human influenza viruses, including genes derived from human influenza virus strains. We believed that these emerging strains would certainly have a major impact on the pigs and human public health, but we could not know specifically how it affected, it required more close monitoring.

It is well known that influenza virus genomes could escape host preexisting immunity by antigenic drift or antigenic shift, resulting in influenza outbreak in animals and even humans [6,53,54]. A recent study has revealed that the 2009H-like, TR-like, CS-like, and EA-like viruses were co-circulating in pigs in southern China with relatively, and 2009H-like viruses might have been maintained in pigs for a period of time and will likely become established in pigs [55]. With the evolution of swine viruses and development of pork industry, in-depth and larger-scale geographic region studies on H1N1 subtype swine influenza viruses in mainland China are still required, to comprehend and monitor the variation, prevalence, transmission, potential hazard of H1N1 subtype swine influenza viruses.

## Conclusions

In conclusion, our study has demonstrated that phylogenetic diversity and complexity existed in H1N1 SIVs in mainland China. Moreover, they were closely related to other subtype influenza viruses, especially human influenza viruses. Through our research, we identified and unified all lineages and genotypes of H1N1 SIVs in mainland China. Novel lineages and genotypes of H1N1 SIVs appeared in mainland China, raising the possibility of generating novel H1N1 SIVs with the potential to infect humans. We provided the essential information to support for further study and confirmation on the genetic and evolutionary characteristics of H1N1 SIVs. Close surveillance should be put into practice about novel lineages and genotypes of H1N1 SIVs in mainland China.

## Methods

### H1N1 subtype swine influenza virus sequences

H1N1 subtype swine influenza virus sequences used in this study were obtained from the NCBI Influenza Virus Resource Database (http://ncbi.nlm.nih.gov/genomes/FLU/FLU.html), till December 27, 2011. Full-length only sequences were collected, containing the complete ORF (Additional file 1: Table S1). BLASTs [56] were conducted on H1N1 subtype swine influenza virus sequences to identify related reference viruses, consisting of viruses isolated from swine, avian, and human.

### Phylogenetic analysis

All the around influenza virus sequences alignments were performed using the software of Clustal X [57] and the genetic distance among the representative sequences were calculated using the model of Kimura-2-Parameter using the software MEGA 5.0 [58]. To estimate the genetic diversity and the level of gene reassortment, phylogenetic trees were constructed for each genomic segment independently. The unrooted phylogenetic trees were constructed for genomic segments using the software MEGA 5.0 with neighboring-joining method [58]. Bootstrap values were calculated out of 1000 replicates.

### Genotypic analysis

Genotypic analysis of H1N1 subtype swine influenza viruses in mainland China was performed systematically for each of the eight gene segments based on the distribution of lineages in phylogenetic trees. Virus genotypes were defined by gene phylogeny. The distinct phylogenetic lineage with bootstrap support of ≥70% indicated a common origin. Viruses only with the classical swine (CS) lineage were designated genotype CS, and viruses with the Eurasian avian-like (EA) lineage and the 2009 human (2009H) lineage were designated genotype EA-2009H. The other genotypes were like this. Genotypes of all H1N1 subtype swine influenza viruses in mainland China analyzed were summarized in Figure 9.

### Ethical approval

All of the animal slaughter experiments were conducted in accordance with the guidelines of Guangdong Province on the Review of Welfare and Ethics of Laboratory Animals approved by the Guangdong Province Administration Office of Laboratory Animals (GPAOLA). All animal procedures were conducted under the protolcol (SCAU-AEC-2010-0416) approved by the Animal Ethics Committee of South China Agricultural University.

## Competing interests

The authors declare that they have no competing interests.

## Authors’ contributions

YL and JW carried out most of the experiments and wrote the manuscript, and should be considered as first authors. QX critically revised the manuscript and the experiment design. JJ, SC, CX, JM, and YB helped with the experiment. All of the authors read and approved the final version of the manuscript.

## Supplementary Material

Additional file 1: Table S1 GenBank accession numbers of gene segments of H1N1 swine influenza viruses isolated in China mainland from 1992 to 2011. Click here for file
