# Supplementary material for: Phylogenetic diversity and genotypic complexity of H1N1 subtype swine influenza viruses isolated in Mainland China
Source: Virol J. 2012 Nov 26;9:289. doi: 10.1186/1743-422X-9-289 (PMC3585526; doi:10.1186/1743-422X-9-289)
Supplement: Additional file 1: Table S1 — GenBank accession numbers of gene segments of H1N1 swine influenza viruses isolated in China mainland from 1992 to 2011. [file 1743-422X-9-289-S1.docx]

Table s1. GenBank accession numbers of gene segments of H1N1 swine influenza viruses isolated in China mainland from 1992 to 2011.

| **Virus Name** | **PB2** | **PB1** | **PA** | **HA** | **NP** | **NA** | **M** | **NS** |
| --- | --- | --- | --- | --- | --- | --- | --- | --- |
| A/swine/Zhejiang/1/2007(H1N1) | FJ415615 | FJ415614 | FJ415616 | FJ415610 | FJ415617 | FJ415611 | FJ415612 | FJ415613 |
| A/swine/Tianjin/01/2004(H1N1) | EU004447 | EU004446 | EU004445 | EU004444 | EU004443 | EU004442 | EU004440 | EU004441 |
| A/swine/Shanghai/3/2005(H1N1) | FJ789830 | FJ789831 | FJ789838 | FJ789832 | FJ789833 | FJ789834 | FJ789835 | FJ789836 |
| A/swine/Shanghai/2/2005(H1N1) | FJ789824 | FJ789825 | FJ789826 | EU502885 | FJ789828 | FJ789827 | FJ789829 | FJ789837 |
| A/swine/Shanghai/1/2005(H1N1) | EU502892 | EU502891 | EU502890 | EU502884 | EU502887 | EU502888 | EU502886 | EU502889 |
| A/swine/Shandong/692/2008(H1N1) | FJ536837 | FJ536836 | FJ536835 | FJ536830 | FJ536833 | FJ536832 | FJ536831 | FJ536834 |
| A/swine/Shandong/62/2008(H1N1) | FJ536799 | FJ536798 | FJ536797 | FJ536792 | FJ536795 | FJ536794 | FJ536793 | FJ536796 |
| A/swine/Shandong/443/2008(H1N1) | FJ536829 | FJ536828 | FJ536827 | FJ536818 | FJ536825 | FJ536824 | FJ536823 | FJ536826 |
| A/swine/Shandong/327/2009(H1N1) | GU086070 | GU086071 | GU086072 | GU086073 | GU086074 | GU086075 | GU086076 | GU086077 |
| A/swine/Shandong/275/2009(H1N1) | GU086062 | GU086063 | GU086064 | GU086065 | GU086066 | GU086067 | GU086068 | GU086069 |
| A/swine/Shandong/187/2008(H1N1) | FJ536809 | FJ536808 | FJ536807 | FJ536802 | FJ536805 | FJ536804 | FJ536803 | FJ536806 |
| A/swine/Shandong/128/2008(H1N1) | FJ536801 | FJ536800 | FJ536786 | FJ536781 | FJ536784 | FJ536783 | FJ536782 | FJ536785 |
| A/swine/Shandong/1123/2008(H1N1) | HM176665 | GU646029 | GU646035 | GU646030 | GU646031 | GU646032 | GU646033 | GU646034 |
| A/swine/Shandong/1112/2008(H1N1) | GU646021 | GU646022 | GU646028 | GU646023 | GU646024 | GU646025 | GU646026 | GU646027 |
| A/swine/Shandong/1012/2008(H1N1) | FJ536841 | FJ536840 | FJ536839 | FJ536819 | FJ536822 | FJ536821 | FJ536820 | FJ536838 |
| A/swine/Shandong/101/2008(H1N1) | GU086054 | GU086055 | GU086056 | GU086057 | GU086058 | GU086059 | GU086060 | GU086061 |
| A/swine/Nanchang/F9/2010(H1N1) | JF275932 | JF275931 | JF275930 | JF275925 | JF275928 | JF275927 | JF275926 | JF275929 |
| A/swine/Nanchang/6/2010(H1N1) | JF275948 | JF275947 | JF275946 | JF275941 | JF275944 | JF275943 | JF275942 | JF275945 |
| A/swine/Nanchang/5/2010(H1N1) | JF275940 | JF275939 | JF275938 | JF275933 | JF275936 | JF275935 | JF275934 | JF275937 |
| A/swine/Nanchang/3/2010(H1N1) | JF275924 | JF275923 | JF275922 | JF275917 | JF275920 | JF275919 | JF275918 | JF275921 |
| A/swine/Liaoning/32/2006(H1N1) | HM754645 | HM754646 | HM754647 | HM754648 | HM754649 | HM754650 | HM754651 | HM754652 |
| A/swine/Jiangsu/s16/2011(H1N1) | JF820282 | JF820283 | JF820284 | JF820285 | JF820286 | JF820287 | JF820288 | JF820289 |
| A/swine/Jiangsu/s15/2011(H1N1) | JF820274 | JF820275 | JF820276 | JF820277 | JF820278 | JF820279 | JF820280 | JF820281 |
| A/swine/Hubei/104/2009(H1N1) | CY091766 | CY091767 | CY091768 | CY091769 | CY091770 | CY091771 | CY091772 | CY091773 |
| A/swine/Hubei/101/2009(H1N1) | CY083005 | CY083006 | CY083007 | CY083008 | CY083009 | CY083010 | CY083011 | CY083012 |
| A/swine/Hubei/03/2009(H1N1) | CY091742 | CY091743 | CY091744 | CY091745 | CY091746 | CY091747 | CY091748 | CY091749 |
| A/swine/Hubei/01/2009(H1N1) | CY091734 | CY091735 | CY091736 | CY091737 | CY091738 | CY091739 | CY091740 | CY091741 |
| A/swine/Henan/01/2006(H1N1) | EU004455 | EU004454 | EU004453 | EU004452 | EU004451 | EU004450 | EU004449 | EU004448 |
| A/swine/Guangxi/1/2011(H1N1) | JN222372 | JN222379 | JN222378 | JN222373 | JN222376 | JN222374 | JN222375 | JN222377 |
| A/swine/Guangdong/L6/2009(H1N1) | HQ880611 | HQ880612 | HQ880613 | HQ880614 | HQ880615 | HQ880616 | HQ880617 | HQ880618 |
| A/swine/Guangdong/L3/2009(H1N1) | HQ877024 | HQ877025 | HQ877026 | HQ877027 | HQ877028 | HQ877029 | HQ877030 | HQ877031 |
| A/swine/Guangdong/94/2009(H1N1) | JN374994 | JN375030 | JN375067 | JN375102 | JN375139 | JN375175 | JN375211 | JN375246 |
| A/swine/Guangdong/628/2006(H1N1) | GU086046 | GU086047 | GU086048 | GU086049 | GU086050 | GU086051 | GU086052 | GU086053 |
| A/swine/Guangdong/611/2006(H1N1) | GU086038 | GU086039 | GU086040 | GU086041 | GU086042 | GU086043 | GU086044 | GU086045 |
| A/swine/Guangdong/50/2010(H1N1) | JN375010 | JN375036 | JN375082 | JN375108 | JN375144 | JN375180 | JN375216 | JN375252 |
| A/swine/Guangdong/446/2006(H1N1) | GU086030 | GU086031 | GU086032 | GU086033 | GU086034 | GU086035 | GU086036 | GU086037 |
| A/swine/Guangdong/34/2006(H1N1) | CY089864 | CY089865 | CY089866 | CY089867 | CY089868 | CY089869 | CY089870 | CY089871 |
| A/swine/Guangdong/33/2006(H1N1) | GU086006 | GU086007 | GU086008 | GU086009 | GU086010 | GU086011 | GU086012 | GU086013 |
| A/swine/Guangdong/322/2006(H1N1) | GU086022 | GU086023 | GU086024 | GU086025 | GU086026 | GU086027 | GU086028 | GU086029 |
| A/swine/Guangdong/297/2010(H1N1) | JN375015 | JN375041 | JN375087 | JN375113 | JN375149 | JN375185 | JN375221 | JN375257 |
| A/swine/Guangdong/294/2010(H1N1) | JN375014 | JN375040 | JN375086 | JN375112 | JN375148 | JN375184 | JN375220 | JN375256 |
| A/swine/Guangdong/286/2010(H1N1) | JN375013 | JN375039 | JN375085 | JN375111 | JN375147 | JN375183 | JN375219 | JN375255 |
| A/swine/Guangdong/278/2010(H1N1) | JN375012 | JN375038 | JN375084 | JN375110 | JN375146 | JN375182 | JN375218 | JN375254 |
| A/swine/Guangdong/275/2010(H1N1) | JN375011 | JN375037 | JN375083 | JN375109 | JN375145 | JN375181 | JN375217 | JN375253 |
| A/swine/Guangdong/221/2009(H1N1) | JN374999 | JN375035 | JN375071 | JN375107 | JN375143 | JN375179 | JN375215 | JN375251 |
| A/swine/Guangdong/213/2009(H1N1) | JN374998 | JN375034 | JN375070 | JN375106 | JN375142 | JN375178 | JN375214 | JN375250 |
| A/swine/Guangdong/176/2009(H1N1) | JN374997 | JN375033 | JN375069 | JN375105 | JN375141 | JN375177 | JN375213 | JN375249 |
| A/swine/Guangdong/1624/2010(H1N1) | JN375024 | JN375065 | JN375101 | JN375137 | JN375168 | JN375196 | JN375240 | JN375268 |
| A/swine/Guangdong/1623/2010(H1N1) | JN375023 | JN375064 | JN375099 | JN375136 | JN375167 | JN375204 | JN375239 | JN375276 |
| A/swine/Guangdong/1619/2010(H1N1) | JN375022 | JN375063 | JN375098 | JN375135 | JN375166 | JN375203 | JN375238 | JN375275 |
| A/swine/Guangdong/1617/2010(H1N1) | JN375021 | JN375061 | JN375100 | JN375134 | JN375165 | JN375202 | JN375237 | JN375274 |
| A/swine/Guangdong/1616/2010(H1N1) | JN375020 | JN375062 | JN375097 | JN375133 | JN375164 | JN375201 | JN375236 | JN375273 |
| A/swine/Guangdong/1613/2010(H1N1) | JN375019 | JN375060 | JN375096 | JN375132 | JN375163 | JN375200 | JN375235 | JN375272 |
| A/swine/Guangdong/1611/2010(H1N1) | JN375018 | JN375059 | JN375095 | JN375131 | JN375162 | JN375199 | JN375234 | JN375271 |
| A/swine/Guangdong/1605/2010(H1N1) | JN375017 | JN375058 | JN375094 | JN375130 | JN375161 | JN375198 | JN375233 | JN375270 |
| A/swine/Guangdong/1604/2010(H1N1) | JN375016 | JN375057 | JN375093 | JN375129 | JN375160 | JN375197 | JN375232 | JN375269 |
| A/swine/Guangdong/1437/2010(H1N1) | JN375009 | JN375051 | JN375081 | JN375128 | JN375159 | JN375195 | JN375227 | JN375267 |
| A/swine/Guangdong/1436/2010(H1N1) | JN375008 | JN375050 | JN375080 | JN375127 | JN375158 | JN375194 | JN375228 | JN375266 |
| A/swine/Guangdong/1434/2010(H1N1) | JN375007 | JN375049 | JN375079 | JN375126 | JN375157 | JN375193 | JN375226 | JN375265 |
| A/swine/Guangdong/1430/2010(H1N1) | JN375006 | JN375048 | JN375078 | JN375125 | JN375156 | JN375192 | JN375225 | JN375264 |
| A/swine/Guangdong/1425/2010(H1N1) | JN375005 | JN375047 | JN375077 | JN375124 | JN375155 | JN375191 | JN375224 | JN375263 |
| A/swine/Guangdong/1409/2010(H1N1) | JN375004 | JN375046 | JN375076 | JN375123 | JN375154 | JN375190 | JN375231 | JN375262 |
| A/swine/Guangdong/1408/2010(H1N1) | JN375003 | JN375045 | JN375075 | JN375122 | JN375153 | JN375189 | JN375230 | JN375261 |
| A/swine/Guangdong/1397/2010(H1N1) | JN375002 | JN375044 | JN375074 | JN375121 | JN375152 | JN375188 | JN375229 | JN375260 |
| A/swine/Guangdong/1367/2010(H1N1) | JN375001 | JN375043 | JN375073 | JN375120 | JN375151 | JN375187 | JN375223 | JN375259 |
| A/swine/Guangdong/1361/2010(H1N1) | JN375000 | JN375042 | JN375072 | JN375119 | JN375150 | JN375186 | JN375222 | JN375258 |
| A/swine/Guangdong/114/2009(H1N1) | JN374996 | JN375032 | JN375068 | JN375104 | JN375140 | JN375176 | JN375212 | JN375248 |
| A/swine/Guangdong/11/2009(H1N1) | HM145745 | HM145744 | HM145743 | HM135403 | HM145742 | HM145741 | HM145746 | HM145747 |
| A/swine/Guangdong/109/2006(H1N1) | GU086014 | GU086015 | GU086016 | GU086017 | GU086018 | GU086019 | GU086020 | GU086021 |
| A/swine/Guangdong/106/2009(H1N1) | JN374995 | JN375031 | JN375066 | JN375103 | JN375138 | JN375174 | JN375210 | JN375247 |
| A/swine/Guangdong/103/2002(H1N1) | GQ422382 | GQ422383 | GQ422384 | GQ422385 | GQ422386 | GQ422387 | GQ422388 | GQ422389 |
| A/swine/Guangdong/1/2010(H1N1) | HM189301 | HM189302 | HM189303 | HM189308 | HM189305 | HM189306 | HM189307 | HM189304 |
| A/swine/Guangdong/09/2009(H1N1) | HM210859 | HM210858 | HM210857 | HM210852 | HM210855 | HM210854 | HM210853 | HM210856 |
| A/swine/Guangdong/09/2008(H1N1) | HM215174 | HM215173 | HM215172 | HM215167 | HM215170 | HM215169 | HM215168 | HM215171 |
| A/swine/Guangdong/07/2009(H1N1) | HM210867 | HM210866 | HM210865 | HM210860 | HM210863 | HM210862 | HM210861 | HM210864 |
| A/swine/Guangdong/07/2008(H1N1) | HM223593 | HM223592 | HM223591 | HM223586 | HM223589 | HM223588 | HM223587 | HM223590 |
| A/swine/Guangdong/06/2009(H1N1) | HM215158 | HM215157 | HM215156 | HM215151 | HM215154 | HM215153 | HM215152 | HM215155 |
| A/swine/Guangdong/05/2009(H1N1) | HM215166 | HM215165 | HM215164 | HM215159 | HM215162 | HM215161 | HM215160 | HM215163 |
| A/swine/Guangdong/02/2008(H1N1) | HM223601 | HM223600 | HM223599 | HM223594 | HM223597 | HM223596 | HM223595 | HM223598 |
| A/swine/Guangdong/01/2008(H1N1) | CY091726 | CY091727 | CY091728 | CY091729 | CY091730 | CY091731 | CY091732 | CY091733 |
| A/swine/Fujian/58/2008(H1N1) | FJ536791 | FJ536790 | FJ536789 | FJ536778 | FJ536787 | FJ536780 | FJ536779 | FJ536788 |
| A/swine/Fujian/204/2007(H1N1) | FJ536816 | FJ536817 | FJ536815 | FJ536810 | FJ536813 | FJ536812 | FJ536811 | FJ536814 |
| A/swine/Fujian/0325/2008(H1N1) | GU646014 | GU646015 | HM176664 | GU646016 | GU646017 | GU646018 | GU646019 | GU646020 |
| A/swine/Beijing/26/2008(H1N1) | FJ536777 | FJ536776 | FJ536775 | FJ536770 | FJ536773 | FJ536772 | FJ536771 | FJ536774 |
| A/swine/Beijing/21/2008(H1N1) | FJ536767 | FJ536769 | FJ536766 | FJ536762 | FJ536764 | FJ536763 | FJ536768 | FJ536765 |
| A/swine/Shandong/N1/2009(H1N1) | / | / | / | JF275949 | / | JF275950 | / | / |
| A/swine/Hubei/S1/2009(H1N1) | / | / | / | GU121819 | / | GU121820 | / | / |
| A/swine/Guangdong/711/2001(H1N1) | / | / | / | AY852271 | / | / | / | / |
| A/swine/Guangdong/3/2005(H1N1) | / | / | / | / | / | / | / | EF140698 |
| A/swine/Guangdong/2/2009(H1N1) | / | / | / | GQ452272 | / | GQ452273 | / | / |
| A/swine/Guangdong/2/2001(H1N1) | / | / | / | DQ058215 | / | / | / | / |
| A/swine/Guangdong/1/2005(H1N1) | / | / | / | FJ536842 | / | / | / | / |
| A/swine/Beijing/47/1991(H1N1) | / | / | / | U46783 |  | / | / | / |
| A/swine/Beijing/94/1991(H1N1) | / | / | / | / | U49091 | / | / | / |
